# First insights into the syntrophic acetate-oxidizing bacteria – a genetic study

**DOI:** 10.1002/mbo3.50

**Published:** 2012-12-13

**Authors:** Bettina Müller, Li Sun, Anna Schnürer

**Affiliations:** Department of Microbiology, Uppsala BioCenter, Swedish University of Agricultural SciencesUppsala, SE 750 07, Sweden

**Keywords:** Acetogens, 10-formyltetrahydrofolate synthetase, hydrogen production, methanogenesis, syntrophic acetate oxidation, Wood–Ljungdahl pathway

## Abstract

Syntrophic acetate-oxidizing bacteria have been identified as key organisms for efficient biogas production from protein-rich materials. They normally grow as lithotrophs or heterotrophs, producing acetate through the Wood–Ljungdahl pathway, but when growing in syntrophy with methanogens, they reportedly reverse this pathway and oxidize acetate to hydrogen and carbon dioxide. However, the biochemical and regulatory mechanisms behind the shift and the way in which the bacteria regain energy remain unknown. In a genome-walking approach, starting with degenerated primers, we identified those gene clusters in *Syntrophaceticus schinkii*, *Clostridium ultunense*, and *Tepidanaerobacter acetatoxydans* that comprise the formyltetrahydrofolate synthetase gene (*fhs*), encoding a key enzyme of the Wood–Ljungdahl pathway. We also discovered that the latter two harbor two *fhs* alleles. The *fhs* genes are phylogenetically separated and in the case of *S. schinkii* functionally linked to sulfate reducers. The *T. acetatoxydans* *fhs*1 cluster combines features of acetogens, sulfate reducers, and carbon monoxide oxidizers and is organized as a putative operon. The *T. acetatoxydans* *fhs*2 cluster encodes Wood–Ljungdahl pathway enzymes, which are also known to be involved in C1 carbon metabolism. Isolation of the enzymes illustrated that both formyltetrahydrofolate synthetases of *T. acetatoxydans* were functionally active. However, only *fhs*1 was expressed, confirming bidirectional usage of the pathway.

## Introduction

Syntrophic acetate oxidation (SAO) has been shown to occur in anaerobic environments such as lake sediments (Nüsslein et al. 2001), oil reservoirs (Nazina et al. 2006), and nutrient-enriched soils (Chauhan and Ogram 2006). Moreover, SAO has been shown to occur in biogas processes (Zinder and Koch 1984; Schnürer et al. 1999; Karakashev et al. 2005, 2006; Schnürer and Nordberg 2008). In general, methane in biogas processes is considered to mainly result from the action of aceticlastic methanogens (Zinder 1984). However, with increasing ammonia levels, released during the degradation of protein-rich materials, this group of organisms is inhibited and acetate is instead oxidized to H_2_ and CO_2_ by syntrophic acetate-oxidizing bacteria (SAOB), thermodynamically driven by the H_2_ consumption of hydrogenotrophic methanogens generating methane (Schnürer and Nordberg 2008; Westerholm et al. 2012). Other factors such as acetate concentration, operational parameters, and microbial community structures have also been considered to influence the acetate conversion pathway (Karakashev et al. 2005, 2006). In addition to previous investigations, a recent study using samples from nine randomly selected large-scale digesters in Sweden revealed that methane is generated through SAO rather than aceticlastic methanogenesis (unpublished). Thus, SAO appears to be of much greater importance for the biogas process than assumed previously and therefore aceticlastic methanogenesis can no longer be considered the main pathway.

**Figure 1:**
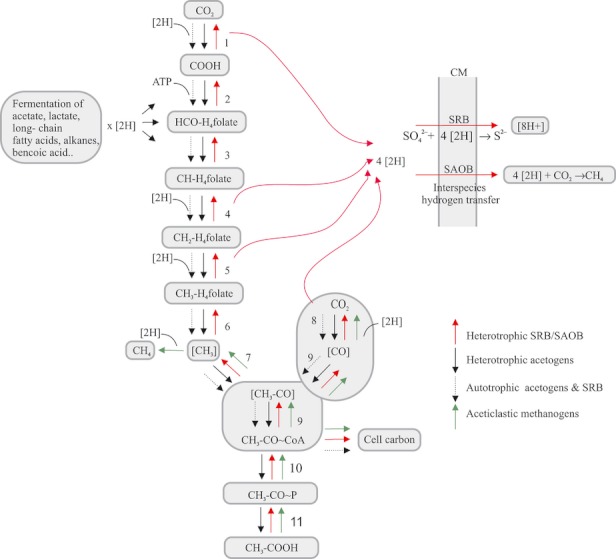
The Wood–Ljungdahl pathway used by acetogens, sulfate-reducing bacteria (SRB), aceticlastic methanogens, and syntrophic acetate-oxidizing bacteria (SAOB). In the methyl branch, free CO_2_ becomes reduced by formate dehydrogenase (1) to formate, which is subsequently attached to tetrahydrofolate in an ATP-dependent reaction catalyzed by *N*^10^-formyltetrahydrofolate synthetase (2). The *N*^10^-tetrahydrofolate-bound formyl group is further reduced via *N*^5^,*N*^10^-methenyl- and *N*^5^,*N*^10^-methylene- to *N*^5^-methyl- by the activities of *N*^5^,*N*^10^-methenyltetrahydrofolate cyclohydrolase (3), *N*^5^,*N*^10^-methylenetetrahydrofolate dehydrogenase (4), and *N*^5^,*N*^10^-methylenetetrahydrofolate reductase (5). The methyl group is transferred by a methyltransferase (6) to a corrinoid enzyme (7), which interacts with the carbonyl branch. Within the carbonyl branch, a second CO_2_ molecule is reduced to an enzyme-bound carbonyl group by a bifunctional enzyme consisting of a carbon monoxide dehydrogenase subunit (CODH) (8), and an acetyl-CoA synthase subunit (ACS). The ACS (9) function finally produces an acetyl moiety from the bound methyl and carbonyl groups, which is subsequently attached to Coenzyme A. Acetyl-CoA can either be incorporated into cell material or converted to acetate via phosphotransacetylase (10) and acetate kinase (11) forming an ATP. CM, cytoplasmic membrane.

Decades of research have focused on aceticlastic methanogens and have neglected SAOB. Therefore, only a few species of SAOB have been isolated and little is known about their physiology and biochemistry. To date, four isolates belonging to the physiological group of acetogens have been characterized and three of these, introduced as *Clostridium ultunense* (Schnürer et al. 1996), *Tepidanaerobacter acetatoxydans* (Westerholm et al. 2011b), and *Syntrophaceticus schinkii* (Westerholm et al. 2010), were isolated in our laboratory. The fourth species, the thermophilic *Thermacetogenium phaeum*, was isolated by Hattori et al. (2000). It has been suggested that the mechanism for converting acetate syntrophically occurs by an oxidative Wood–Ljungdahl (W–L) pathway (Lee and Zinder 1988; Schnürer et al. 1997; Hattori et al. 2005), as used by certain sulfate reducers and aceticlastic methanogens (Fig. 1). However, in this case, the process is highly endergonic (ΔG°′ = +95 kJ/mol) when using protons as electron acceptor. It has been postulated that the immediate consumption of hydrogen gas by the methanogenic partner keeps the hydrogen partial pressure low enough to make the reaction sufficiently exergonic (ΔG°′ = −36 kJ/mol) and allows ATP synthesis (Schink 1997). The W–L pathway, used by SAOB in a reductive way when growing heterotrophically, can be viewed as a series of reactions resulting in the reduction of two molecules of CO_2_ to a bound methyl and carbonyl group, which finally form the acetyl moiety of acetyl-CoA (Fig. 1). It is currently unknown whether there are any differences between the pathways operating in one direction (forming acetate) and the other (consuming acetate). Moreover, other acetogens such as *Moorella thermoacetica*, *Thermoanaerobacter kivui*, or *Acetobacterium woodii* seem unable to use this pathway in an oxidative way and to grow syntrophically with hydrogenotrophic methanogens (Winter and Wolfe 1980; Zinder and Koch 1984; Cord-Ruwisch et al. 1988). Thus, SAO seems not to be a common physiological feature of acetogens.

To further understand the activity of SAOB in different environments, it is critical to understand the respective biochemical and regulatory mechanisms behind acetate production and acetate oxidation. To start with, we identified the *fhs* genes of *T. acetatoxydans*, *S. schinkii*, and *C. ultunense* encoding formyltetrahydrofolate synthetase, a key enzyme of the W–L pathway, by developing a new degenerated primer pair. We then analyzed the gene structure adjacent to the *fhs* genes by genome walking and investigated *fhs* mRNA expression in a syntrophic coculture. We also cloned two of the *fhs* genes and determined the specific activities of the gene products after purification. Thus, this article presents the first steps toward understanding the thermodynamically unique SAO pathway.

## Experimental Procedures

### Bacterial strains and growing conditions

*Clostridium ultunense* strain Esp (JCM 16670), *S. schinkii* strain Sp3 (DSM 21860), and *T. acetatoxydans* strain ReI (DSM 21804) were cultivated at 37°C in basal medium modified as described by Schnürer et al. (1996) in the presence of 10 mmol/L lactate, 10 mmol/L betaine, and 10 mmol/L glucose, respectively. A coculture consisting of *C. ultunense*, *S. schinkii*, *T. acetatoxydans*, and the hydrogenotrophic *Methanoculleus* sp. MAB1 was cultivated in the same medium supplemented with 10 mmol/L acetate and 0.2 mol/L NH_4_Cl. *Thermacetogenium phaeum* strain PB (DSM 12270) was obtained from Deutsche Sammlung von Mikroorganismen und Zellkulturen (DSMZ) (Braunschweig, Germany) and cultivated as recommended by DSMZ. The *C. ultunense* strain BST deposited in the DSMZ has been identified as impure and was therefore replaced in this study by strain Esp, newly isolated in our laboratory (Westerholm et al. 2010). The *Escherichia coli* strains JM109 (Promega, Madison, WI) and BL21 (DE3) (Novagen, Merck KGaA, Darmstadt, Germany) were grown at 37°C in Luria–Bertani (LB) medium.

### DNA isolation

Genomic DNA was isolated and purified using the DNeasy Blood & Tissue Kit from Qiagen (Hilden, Germany) and concentrated by salt/ethanol precipitation according to Sambrook et al. (1989). DNA purity was confirmed by amplifying and sequencing the respective 16SRNA gene using PuReTaq Ready-to-go PCR beads (GE Healthcare, Buckinghamshire, U.K.) and the universal primer pair EC9-26f (GAGTTTGATCMTGGCTCA, modified “fD2” and 926r; Weisburg et al. 1991; Jernberg and Jansson 2002). To check purity more sensitively, the amplified 16sRNA gene in the case of *T. acetatoxydans* and *C. ultunense* was purified (QIAquick PCR Purification Kit, Qiagen), cloned into the pGEMTeasy vector (Promega), and analyzed by colony PCR following the manufacturer's instructions using *E. coli* JM109 as host strain. A total of 40 clones each were screened for alien DNA by sequencing 16SRNA. Plasmids were isolated from LB-grown cultures using the QIAprep Spin Miniprep Kit from Qiagen according to the manufacturer's instructions.

### RNA isolation

RNA was isolated from exponentially growing pure cultures or cocultures using the RNeasy Mini Kit from Qiagen according to the manufacturer's instructions with the following modification: Cells were disrupted by bead beating (speed 5.5 for 40 sec) using a homogenizer and glass beads from the FastDNA soil kit from MP Biomedicals (France). Genomic DNA was removed using DNaseI from Fermentas (Germany), as suggested by the manual. Complete DNA digest was checked by PCR using PuReTaq Ready-to-go PCR beads and gene-specific primers as listed in Table 1 (application: mRNA expression). PCR included an initial denaturation at 95°C for 3 min, 35 cycles of denaturation at 95°C for 30 sec, annealing at 60°C for 30 sec, and elongation at 72°C for 40 sec, followed by a final elongation step at 72°C for 7 min.

**Table 1:** Primers used for genome walking, primer walking, and mRNA expression studies

Primer name	Sequence^1^	Organism/source	Gene^2^	Application
I ESP GSP1 up	AAATCCATCTTCTCTAGGTACACCGTT	*Clostridium ultunense* strain Esp	*fhs*1	Upstream walk
II ESP GSP1 down	CTGAAGAACTAGGTTCAGTAGCAGTAC	*C. ultunense* strain Esp	*fhs*1	Downstream walk
III ESP GSP2 down	GAGACGGTGGAATCGAATTAGCTAAGA	*C. ultunense* strain Esp	*fhs*1	Downstream walk
I 2.fhs ESP GSP1 up	CTCCTCCATGGGAACCACCTGTGAGTA	*C. ultunense* strain Esp	*fhs*2	Upstream walk
II 2.fhs ESP GSP1-3 up	GGTGGATCTTCGCTTTATATTTTCCGT	*C. ultunense* strain Esp	*fhs*2	Upstream walk
III 2.fhs ESP GSP1 down	CGGAAGGGGCCATGGCCCTTTTGTTAA	*C. ultunense* strain Esp	*fhs*2	Downstream walk

I Sp3 GSP1 up	TGTGTGCGTTCATGATGTCATTGATGT	*Syntrophaceticus schinkii* strain Sp3	*fhs*	Upstream walk
II Sp3 GSP2 up	AGCGGCAGACCCTTTAATGTTCATAGT	*S. schinkii* strain Sp3	*fhs*	Upstream walk
IV Sp3 GSP1 down	TTGATGGCCATTTTGGCTGTGGCAAAA	*S. schinkii* strain Sp3	*fhs*	Downstream walk
V Sp3 GSP2 down	AACAAGAAGGGTGAGCCGGTTACAACA	*S. schinkii* strain Sp3	*fhs*	Downstream walk
III Sp3 GSP1-1 up	ATAACACCAGAGGAAGGCAAGACCTAT	*S. schinkii* strain Sp3	*fhs*	Upstream walk
VI Sp3 GSP1-1 down	TGCACTGGAACTGGCAGATGCTGTTAT	*S. schinkii* strain Sp3	*fhs*	Downstream walk
VII Sp3 A5 downstream primer	TTCTGCAAGCATAACCTGCAGCACTTT	*S. schinkii* strain Sp3	*fhs*	Primer walking

I first primer upstream GSP1	TTTTCCATCATATGTATAACCAATTAT	*Tepidanaerobacter acetatoxydans* strain ReI	*fhs*1	Upstream walk
II nested primer upstr GSP2	GCAAGCCTATTTTTCAAATCTTCCAT	*T. acetatoxydans* strain ReI	*fhs*1	Upstream walk
III first primer upstr GSP1-2	TTACCGAGCCTTCTGAGTGCATCACCT	*T. acetatoxydans* strain ReI	*fhs*1	Upstream walk
IV nested primer up GSP2-2	TTTGCCTTCACCTGCAGGAGTTGGATT	*T. acetatoxydans* strain ReI	*fhs*1	Upstream walk
V DL2 upstream primer	ATAGCAGGTGCTGCATCAATGCCCTC	*T. acetatoxydans* strain ReI	*fhs*1	Upstream walk
VI GSP1-3up	GTCTGCATCACAGCCGATATGTGTTGA	*T. acetatoxydans* strain ReI	*fhs*1	Upstream walk
VII nested GSP2-3up	TGGCCCGAGGCACCACATTCATCTTTT	*T. acetatoxydans* strain ReI	*fhs*1	Upstream walk
VIII DL1 2.3-kb up primer	TTGTGCAAAGCATGCACTGCTCTAATT	*T. acetatoxydans* strain ReI	*fhs*1	Primer walking
VIV GSP1-4up	CAAAGATAACCGATTCGGCAATCCCAA	*T. acetatoxydans* strain ReI	*fhs*1	Upstream walk
I 2.*fhs* ReI GSP1 down	CATATGATCGGGAGGGTAAGCCCGTTA	*T. acetatoxydans* strain ReI	*fhs*2	Downstream walk
II 2.*fhs* ReI nested GSP2 down	CAAGGTGCCATGGCGGCTCTACTCAAA	*T. acetatoxydans* strain ReI	*fhs*2	Downstream walk
III 2.*fhs* downstr pr (A1/A2)	GCCCTGAAATAACACCCTTTTCATCCA	*T. acetatoxydans* strain ReI	*fhs*2	Primer walking
IV 2.*fhs* GSP1-2 down	CGCGGAGCCCTTAAAGCAATGGCAAAT	*T. acetatoxydans* strain ReI	*fhs*2	Downstream walk
V DL1 (A1) 2.fhs downstr pr5'	CCCTTTGAAGAAATAACTGAGTATTTA	*T. acetatoxydans* strain ReI	*fhs*2	Primer walking
VI DL1 (A1) 2.fhs downstr pr3'	GTGCCAATTCGGCAGTAACTGCAAACT	*T. acetatoxydans* strain ReI	*fhs*2	Primer walking
VII 2.*fhs* ReI GSP1 up	GGGCTTACCCTCCCGATCATATGCTAC	*T. acetatoxydans* strain ReI	*fhs*2	Upstream walk
VIII 2.*fhs* ReI GSP2 up	CTTCGCCAGCCGTTCTTTCAAATCCAT	*T. acetatoxydans* strain ReI	*fhs*2	Upstream walk
VIV 2.*fhs* ReI GSP2-2up	CGACCTTTGCTTTATATTTGCCGTACA	*T. acetatoxydans* strain ReI	*fhs*2	Upstream walk
X 2.*fhs* DL4 upstream pr	TGCCTGACAGCAAGATCATGAATAATT	*T. acetatoxydans* strain ReI	*fhs*2	Primer walking
X first primer down GSP1	AAAGAGAGCTGGCACTGGTTCAAGAAG	*T. acetatoxydans* strain ReI	*fhs*1	Downstream walk
XI nested primer down GSP2	GCAGTGCTTTCCGAGGTTTGGGCAAAA	*T. acetatoxydans* strain ReI	*fhs*1	Downstream walk
XII DL3 downstream primer	AGCACTTACCGGTGCTATCATGACAAT	*T. acetatoxydans* strain ReI	*fhs*1	Primer walking
XIII DL3 downstream primer2	ATGTTCATCGCCCTGTGGTGCAACATA	*T. acetatoxydans* strain ReI	*fhs*1	Primer walking
1.*fhs* locus rev	CCATTACTATGCACAAACCTAAAGCCT	*T. acetatoxydans* strain ReI	*fhs*1	Locus verification
2.*fhs* locus rev	TCAATGGGATCATATTCCGGAGTTACA	*T. acetatoxydans* strain ReI	*fhs*2	Locus verification
1.locus Oxido fw	TTATTTGCGATTATCCGCAGGAAAGAC	*T. acetatoxydans* strain ReI	*fhs*1	Locus verification
2.locus Methyltr fw	ATGATTATTATTGGGGAAAAGATTAAC	*T. acetatoxydans* strain ReI	*fhs*2	Locus verification
Esp 1.*fhs* RNA fw	TAGACCAAACGGTGTACCTA	*C. ultunense* strain Esp	*fhs*1	mRNA expression
Esp 1.*fhs* RNA rev	AAGTAAAGCTACTGCTCCCT	*C. ultunense* strain Esp	*fhs*1	mRNA expression
Esp 2.*fhs* RNA fw	AAAGAAAAACGGAGTGCCTC	*C. ultunense* strain Esp	*fhs*2	mRNA expression
Esp 2.*fhs* RNA rev	CTTTAACAAAAGGGCCATGG	*C. ultunense* strain Esp	*fhs*2	mRNA expression
ReI 1.*fhs* RNA fw	GGCTGCAACAGTATAATAGC	*T. acetatoxydans* strain ReI	*fhs*1	mRNA expression
ReI 1.*fhs* RNA rev	TCTTGACCCCGCCATTATAT	*T. acetatoxydans* strain ReI	*fhs*1	mRNA expression
ReI 2.*fhs* RNA fw	TGTTGTAGCATATGATCGGG	*T. acetatoxydans* strain ReI	*fhs*2	mRNA expression
ReI 2.*fhs* RNA rev	TTACCGAGTTACATCCGTGT	*T. acetatoxydans* strain ReI	*fhs*2	mRNA expression
Sp3 *fhs* RNA fw	CTCGAAAGACGGTTTCTTGA	*S. schinkii* strain Sp3	*fhs*	mRNA expression
Sp3 *fhs* RNA rev	TTGATCGTGTTACGCATCCA	*S. schinkii* strain Sp3	*fhs*	mRNA expression

*T. phaeum* this study fw	CGGACCCACCATGAACATTAAGGGTA	*Thermacetogenium phaeum* strain PB	*fhs*	Authenticity check
*T. phaeum* this study rev	AAGTCGATAACCCAGCCCATTTCCAC	*T. phaeum* strain PB	*fhs*	Authenticity check
*T. phaeum* AB523739 fw	ATCTTGGCTCTAACTACCGGCCTCAA	*T. phaeum* strain PB	*fhs*	Authenticity check
*T. phaeum* AB523739 rev	CTCGTTGCCATCATATCGGCCAGTAT	*T. phaeum* strain PB	*fhs*	Authenticity check

M13 fw	GTAAAACGACGGCCAGTG	Invitrogen		Sequencing
M13 rev	GGAAACAGCTATGACCATG	Invitrogen		Sequencing
Adaptor primer 1 (AP1)	GTAATACGACTCACTATAGGGC	GenomeWalker Kit		Sequencing/walk
Nested Adaptor primer 2 AP2)	ACTATAGGGCACGCGTGGT	GenomeWalker Kit		Sequencing/walk

Primers were designed using Geneious v4.5 and synthesized by Invitrogen (Carlsbad, CA).

1 Sequences are indicated as 5′ to 3′.

2 Gene used to start genome walking or targeted in mRNA expression studies.

### mRNA expression studies

DNAseI-treated RNA samples were reverse transcribed using RevertAid Premium Reverse Transcriptase and random hexamer primers from Fermentas as recommended. cDNA were used directly in PCR as recommended, applying same conditions and primers as mentioned above.

### Construction of DNA libraries

Four different DNA libraries each of *C. ultunense*, *S. schinkii*, and *T. acetatoxydans* were constructed using the Clontech Universal GenomeWalker kit (Clontech Laboratories, CA). According to the manufacturer's instructions, the respective genomic DNA was digested with *Hinc*II (New England Biolabs, Herts, U.K.), *Eco*RV, *Pvu*II, or *Stu*I (Clontech) and subsequently ligated to the GenomeWalker Adaptor resulting in uncloned, adaptor-ligated genomic DNA fragments referred as “libraries.” In addition, *Dra* I libraries were generated of *C. ultunense* and *T. acetatoxydans*. All DNA libraries were stored at −20°C.

### Genome walking

Genome walking was carried out using gene-specific primers (Table 1) targeting the partial *fhs* gene of the respective strain in combination with the AP1/AP2 primer set (Clontech) directed to the adaptor sequence. The simplified touchdown PCR was performed according to the manufacturer's protocol using the Advantage 2 PCR kit (Clontech) or PuReTaq Ready-to-go PCR beads. If necessary, a nested PCR was performed as recommended by Clontech. The fragments obtained ranged in size from 0.4 to 3 kb and were purified using QIAquick PCR Purification Kit or Gel Extraction Kit (Qiagen) before sequencing. Fragments larger than 1.5 kb were cloned into the pGEMTeasy vector as described above. The resulting plasmids were further analyzed by primer walking (Table 1) and/or sequenced by the M13 primer set.

### Verification of *T. acetatoxydans fhs* loci

Accurate genome walking and assembly of the two separately located *fhs* loci were verified by amplification of 5.0 kb (primer combination A), encompassing the 5′ *fhs*1 tail and its upstream region, and 2.1 kb (primer combination B), encompassing the 3′ *fhs*1 tail and its downstream region. The *fhs*2 locus was confirmed in the same way, generating a 1.5-kb fragment (primer combination C) encoding the 5′ *fhs*2 tail and its upstream region and a 4.0-kb fragment (primer combination D) encoding the 3′ *fhs*2 and its downstream region (data not shown). Therefore, a touchdown PCR was performed including an initial denaturation at 95°C for 5 min, 11 cycles of denaturation at 94°C for 60 sec, annealing at 63°C for 60 sec (decreased by 1°C per cycle to 53°C), and elongation at 72°C for 210 sec followed by 32 cycles consisting of 94°C for 60 sec, 53°C for 60 sec, and 72°C for 210 sec, and finalized by 7 min at 72°C. The reaction system consisted of 10 pmol of each primer, 20 ng genomic DNA, and Ready-to-go PCR beads. The primer combinations A–D were set up as follows: (A) 1.locus Oxido fw/nested primer upstream GSP2; (B) First primer downstream GSP1/1.*fhs* locus rev; (C) 2.locus Methyltr fw/2.*fhs* ReI GSP2 up; and (D) 2.fhs ReI nested GSP2 down/2.*fhs* locus rev (primer sequences are listed in Table 1).

### DNA sequencing

Sequencing was performed by Uppsala Genome Center, Sweden using costumer provided primers as listed in Table 1.

### Prediction and annotation of CDS

Sequence assembly, editing, and prediction of coding sequences (CDSs) and open reading frames (ORFs) were achieved by Geneious v5.4 (Drummond et al. 2011). All CDSs were double checked and corrected manually by comparing the predicted protein sequences with the publicly available database of GenBank using BLAST (Altschul et al. 1990). BROM, available online at http://www.softberry.com, was used as the promoter-prediction program. MEME (MEME@ncbi.net) was used for analyzing palindromes and repetitive sequences. Codon usage patterns were analyzed on http://www.geneinfinity.org.

### Tree construction

Multiple sequence alignments were performed using MUSCEL 3.8.31 (Edgar 2004). A maximum-likelihood tree was constructed using PhyML 3.0 (Guindon and Gascuel 2003) choosing the WAD substitution matrix and MEGA5.05 (Tamura et al. 2011). MUSCEL 3.8.31 and PhyML 3.0 are both available on the Mobyle platform of Institut Pasteur.

### Plasmid construction

Plasmid pBM06 was constructed by ligating a PCR fragment encompassing the *fhs*1 gene (encoding FTHFS1) of *T. acetatoxydans* as an *Nde*I/*Bam*HI fragment with the expression vector pET15b (Novagen). Plasmid pBR01 was constructed likewise carrying the *fhs*2 gene (encoding FTHFS2) of *T. acetatoxydans*. A partial digest was performed in the case of *Nde*I due to an internal restriction site. Sequencing revealed a change in genotype (T→C) at bp 425 for *fhs*1 compared with the sequence obtained by genome walking. At this stage of the study, it was not clear which nucleotide reflected reality. However, multiple sequence alignment using 80 randomly picked publicly available formyltetrahydrofolate synthetases (FTHFS) revealed the affected residue (Leu_142_→Ser) to be not conserved and located outside any catalytic site.

### Purification procedures

FTHFS1 and FTHFS2 of *T. acetatoxydans* were purified as N-terminal His-tagged fusion proteins from cell extract of *E. coli* strain BL21 (DE3) (Novagen) harboring plasmid pBB06 or pRB01, respectively. Cells were grown under vigorous shaking in LB medium at 37°C and overexpression of *fhs*1 or *fhs*2 was induced by adding 0.5 mmol/L IPTG at OD 0.3 for 3 h. Cells were harvested and resuspended in 100 mmol/L Tris/HCl, pH 7.5, containing 50 μg/mL DNase (Sigma-Aldrich, Seelze, Germany). Cell extract was recovered by centrifugation after disruption of the cells using a French press. Both recombinant proteins were purified by IMAC immobilized metal-affinity chromatography) using TALON metal-affinity resin from Clontech. The washing buffer contained 0.3 mol/L NaCl in 0.05 mol/L Tris/HCl, pH 7.5. Proteins were eluted with 0.15 mol/L imidazole added to the washing buffer. Elution fractions enriched with the respective protein were pooled and subsequently washed with 0.15 mol/L NaCl in 0.1 mol/L Bis-Tris/HCl, pH 6.9 and concentrated at the same time using Vivaspin 20 concentrator columns (Sartorius Stedim Biotech, cut-off size 10 kDa). All solutions were supplemented with EDTA-free protease inhibitor cocktail (Roche Diagnostics, Mannheim, Germany) as recommended. To keep the proteins soluble at higher concentrations, 5 mmol/L DTT (dithiothreitol) was added to FTHFS 2 and 2 mmol/L DTT to FTHFS 1. Proteins were stored at −20°C.

### FTHFS assay

Enzymatic formylation of *d,l*-tetrahydrofolate was measured at 60°C based on the assay method of Rabinowitz and Pricer (1962). The reaction mixture contained 100 mmol/L sodium formate, 10 mmol/L NH_4_Cl, and 2 mmol/L *d,l*-tetrahydrofolate (Sigma-Aldrich; stabilized with 1 mol/L β-mercaptoethanol at pH 7.5) in 100 mmol/L HEPES, pH 8.3. In order to calculate the specific activities, protein was added in a range from 0.05 to 50 μg/mL. After 5 min preincubation at 60°C, the reaction was started by adding ATP/MgCl_2_ to a final concentration of 5 mmol/L and 10 mmol/L, respectively. Samples were taken after 1, 2, 3, 4, 5, and 10 min, and the reactions were stopped by adding 0.36 N HCl in a ratio of 1:2. HCl converts the product 10-formyltetrahydrofolate to 5,10-methenyltetrahydrofolate (ε_350nm_ = 24,900 L* mol^−1^* cm^−1^), which was measured spectrophotometrically at 350 nm (Infinite M200 plate reader, TECAN) after 10 min. Specific activity was calculated from product absorbency, linearly proportional to time and number of micrograms. All activity measurements were run in triplicate.

### Other analytical procedures

Protein determination was carried out using the protein assay kit from Bio-Rad. SDS-PAGE (SDS-polyacrylamid gel electrophoresis) was performed using precast 4–15% mini-PROTEAN SDS-PAGE gels and the respective equipment purchased from Bio-Rad. Mass spectrometry was carried out by the Department of Medical Biochemistry and Microbiology (Biomedical Center, Uppsala, Sweden) using MALDI/TOF (Ultraflex MALDI TOF, Bruker Daltonics). DNA was visualized by agarose–ethidium bromide electrophoresis using 1.0% or 1.5% agarose gels and 1-kb DNA ladder from Fermentas.

### Nucleotide sequence accession number

The nucleotide sequence data reported in this study were deposited at GenBank with accession numbers as follows: JQ979072 (*T. acetatoxydans fhs*1 locus), JQ979073 (*T. acetatoxydans fhs*2 locus), JQ979075 (*C. ultunense fhs*1 locus); JQ979076 (*C. ultunense fhs*2 locus), JQ979074 (*S. schinkii fhs* locus), and JQ979077 (*T. phaeum* partial *fhs*).

### Design of degenerated FTHFS primers

**Figure 2:**
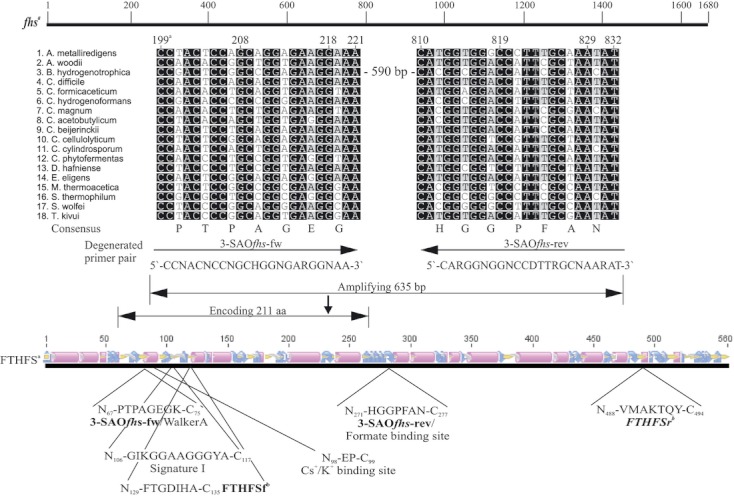
Nucleic acid sequence alignment of the degenerated primer-binding sites exemplified with 18 full-length *fhs* genes. (a) bp and amino acid numbers are based on the *N*^10^-formyltetrahydrofolate synthetase of *Moorella thermoacetica*. (b) FTHFS primer pair designed by Leaphart and Lovell (2001). For further details, see Results section.

Full-length FTHFS sequences of acetogens and nonacetogens including *Alkaliphilus metalliredigens* (CP000724), *Acetobacterium woodii* (AF295701), *Blautia hydrogenotrophica* (ACBZ01000230), *Clostridium difficile* (FN54816), *Clostridium formicaceticum* (AF295702), *Carboxydothermus hydrogenoformans* (CP000141), *Clostridium magnum* (AF295703), *Clostridium acetobutylicum* (AE001437), *Clostridium beijerinckii* (NC009617), *Clostridium cellulolyticum* (CP001348), *Clostridium cylindrosporum* (L12465), *Clostridium phytofermentas* (CP000885), *Desulfitobacterium hafniense* (CP001336), *Eubacterium eligens* (CP001104), *M. thermoacetica* (CP000232), *Symbiobacterium thermophilum* (AP006840), *Syntrophomonas wolfei* (CP000448), *T. kivui* (AF295704), *Anaerostipes caccae* (DS499743), *Bacillus cereus* (EEL11738), *Clostridium acidiurici* (M21507), *Clostridium botulinum* (CP000727), *Clostridium kluyveri* (CP000673), *Clostridium novyi* (CP000382), *Clostridium perfringens* (CP000246), *Clostridium thermocellum* (CP000568), *Clostridium tetani* (AE015927), *Eggerthella lenta* (CP001726), *Eubacterium rectale* (CP001107), *Selenomonas sputigena* (CP002637), *Slackia heliotrinireducens* (CP00168), and *Thermoanaerobacter spec*. (CP000923), as well as partial FTHFS sequences of homoacetogens and sulfate reducers (Leaphart and Lovell 2001; Leaphart et al. 2003; Matsui et al. 2008; Westerholm et al. 2011a), were aligned in Geneious v5.4 with ClustalW. A new primer pair designated 3-SAO*fhs*-fw (CCNACNCCNGCHGGNGARGGNAA) and 3-SAO-rev (ATRTTNGCRAADGGNCCNCCRTG) was designed targeting identified conserved stretches (Fig. 2).

### PCR amplification of *fhs* genes

The primer pair FTHFSf/FTHFSr designed by Leaphart and Lovell (2001) was used in a touchdown PCR as described, but with the annealing temperature decreased down to 53°C. PCR reactions were performed using Ready-to-go PCR beads, 20 ng genomic DNA, and 20 pmole of each primer. Alternatively, PCR conditions were adjusted as described by Lovell and Leaphart (2005) using 49°C and 47°C, respectively, as annealing temperature. The optimized touchdown PCR used for the 3-SAO*fhs*-fw/rev primer pair was performed after an initial melting step at 94°C for 5 min comprising 11 cycles consisting of 60 sec at 94°C, 60 sec at 63°C (decreased by 1°C per cycle to 53°C), and 60 sec at 68°C, followed by 30 cycles at 94°C for 60 sec, 53°C for 60 sec, and 68°C for 60 sec, finalized by 20 min at 68°C. PCR reactions consisted of Ready-to-go PCR beads, 20 ng genomic DNA, and 50 pmole of each primer. The PCR conditions used for the specific *T. phaeum* primer (listed in Table 1) targeting either the partial *fhs* gene identified in this study or the partial *fhs* gene (AB523739) identified by Hori et al. (2011) were as follows: 95°C 5 min; 30 cycles of 94°C for 1 min, 61°C for 1 min, 72°C for 1 min, finalized by 5 min at 72°C. Here, 10 pmole of each primer were used.

## Results

### *Tepidanaerobacter acetatoxydans and Clostridium ultunense* harbor two *fhs* alleles

The FTHFSf/FTHFSr primer pair designed by Leaphart and Lovell (2001) has been used successfully for targeting partial *fhs* species in numerous environmental samples and in pure cultures (see Experimental Procedures section). However, in the case of SAOB, the recommended touchdown PCR generated an amplicon for *C. ultunense*, but not for *T. acetatoxydans*, *S. schinkii*, and *T. phaeum*. After reducing the annealing temperature down to 47°C as suggested by Lovell and Leaphart (2005), an impure amplification was also achieved for *T. acetatoxydans* (data not shown). Hence, based on an alignment of the deduced polypeptide sequences of a total of 27 full-length *fhs* genes (representatives are shown in Fig. 2) and an additional 40 partial *fhs* genes (see Experimental Procedures), a new primer pair was designed (Fig. 2): The forward primer 3-SAO*fhs*-fw shows a degeneracy at seven positions of a total of 23 base pairs and is directed to the WalkerA motif, a common feature of all members of the super family of P-type NTPases (Walker et al. 1982). For the reverse primer 3-SAO*fhs*-rev, a stretch of seven amino acid residues resulting in a sevenfold degenerated 23mer primer was identified as highly conserved within the FTHFS, but with fewer similarities to the P-type NTPases super family. The primer pair amplifies approximately 635 base pairs encoding a polypeptide of 211 amino acid residues encompassing the signature I motif and the cesium/potassium-binding site (Radfar et al. 2000), allowing appropriate allocation of the sequences obtained.

In the case of SAOB, it was possible to generate amplicons from purified genomic DNA of all three strains considered in this study and of *T. phaeum* at the expected size. However, sequencing of the PCR products revealed impure amplification, and the PCR products were therefore cloned and further analyzed. To our surprise, two different partial *fhs* genes of *T. acetatoxydans* and of *C. ultunense* were found. For *S. schinkii* and *T. phaeum*, only one partial *fhs* gene each was identified.

### The covered *fhs* genes are highly phenotypically distinguished

**Figure 3:**
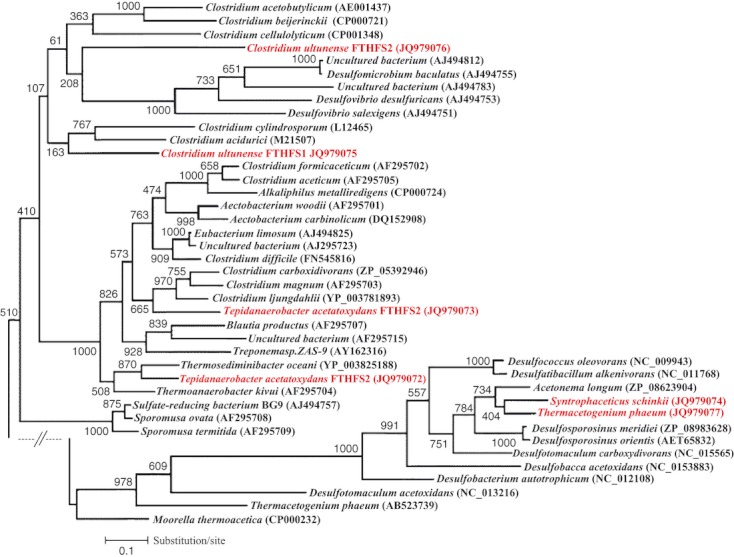
Phylogenetic placement of deduced amino acid sequences of the full-length *fhs* genes of *Clostridium ultunense*, *Syntrophaceticus schinkii*, and *Tepidanaerobacter acetatoxydans*, and the partially recovered *fhs* gene of *Thermacetogenium phaeum*. Positions are highlighted in red. The tree is based on maximum-likelihood alignment. Bootstrap values supported by 1000 replicates are indicated at the nodes if larger than 50%. GenBank accession numbers are given in brackets.

In order to evaluate the FTHFS from SAOB phylogenetically, we completed the adjacent upstream and downstream sequences of the covered partial *fhs* genes by genome walking using adaptor-ligated libraries of *C. ultunense*, *T. acetatoxydans*, and *S. schinkii* in combination with gene-specific primers. Maximum-likelihood tree construction based on the deduced amino acid sequences revealed them to be highly phenotypically distinguished from each other and with partly poor identities to known acetogens. As indicated in the dendrogram (Fig. 3), the FTHFS1 of *T. acetatoxydans* formed a tight group with the recently sequenced acetogen *Thermosediminibacter oceani* isolated from sea floor sediment (Pitluck et al. 2010), showing 85.4% sequence identity (Table 2). Distinctly separated from FTHFS1, the FTHFS2 of *T. acetatoxydans* clustered together with Clostridia species described as autotrophic acetogens. Thus, *Clostridium carboxidivorans* as the nearest relative showed 78.7% sequence identity, followed by *Clostridium ljungdahlii* and *C. magnum* with 78.7% and 77.5% identity, respectively. The FHTFS1 of *C. ultunense* was found to be clustered together with FTHFS amplified from purinolytic *Clostridia*, lacking the W–L pathway. The level of sequence identity between *C. ultunense* and *C. cylindrosporum* and *Clostridium acidurici* was 71.5% and 70.2%, respectively. However, the phylogenetic classification was rather low, with only 16.3% of the bootstrap replicates. The FTHFS2 of *C. ultunense* was substantially outgrouped and showed no similarity to acetogens and purine fermenters or to sulfate reducers. A protein–protein blastp search performed against the nonredundant protein database revealed *Desulfitobacterium metallireducens* as the closest relative, with 66% identity. The FTHFS of both *S. schinkii* and *T. phaeum* were found to be tightly grouped within a cluster representing mainly known sulfate reducers. Although the sequence identity between *T. phaeum* and *S. schinkii* was significantly high (81.4%), the bootstrap value was below 50% because of the truncated polypeptide of *T. phaeum* used in the alignment. The autotrophic acetogen *Acetonema longum* and the sulfate reducer *Desulfosporosinus meridiei* had the closest relative identities, ranging from 80.9% to 83.1% compared with *S. schinkii* and *T. phaeum*, respectively (Table 2).

**Table 2:** Pairwise distance among the different FTHFS obtained and between them and their closest relatives as shown by the phylogenetic tree

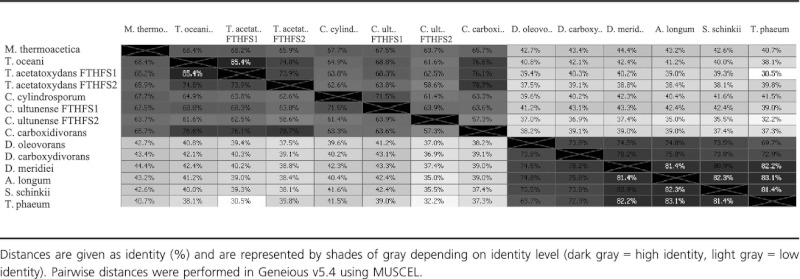

### Two large insertions within FTHFS connect *S. schinkii* and *T. phaeum* with sulfate reducers

**Figure 4:**
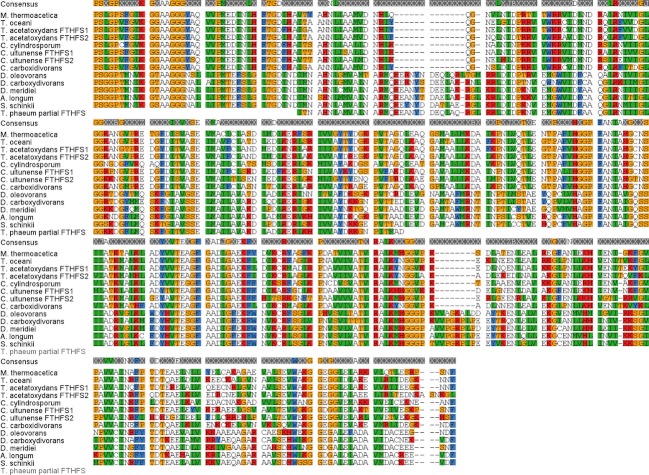
Multiple sequence alignment of the full-length FTHFS of *Tepidanaerobacter acetatoxydans*, *Syntrophaceticus schinkii*, and *Clostridium ultunense*, and the partial FTHFS of *Thermacetogenium phaeum* in comparison with their closest relatives. Only the amino acid residues 100–437 are shown based on the amino acid number of the FTHFS sequence of *Moorella thermoacetica*. Sequence alignment was performed in Geneious v5.4 using MUSCLE.

Multiple sequence alignment revealed two large insertions, which seem to be common to members of the *S. schinkii*/*T. phaeum* cluster (Fig. 4, not all members are shown). Thirteen additional amino acid residues are inserted between residues 152 and 153 referring to the numbering of the FTHFS of *M. thermoacetica*. Another 8-amino acid insertion is located between residues 343 and 344, followed by a 2-amino acid insertion within the N-terminus after residue 15 and two single amino acid insertions after residues 478 and 550, respectively (the latter not shown). Controversially, the recently published partial sequence of *T. phaeum* (AB523739; Hori et al. 2011) fell outside the *S. schinkii*/*T. phaeum* cluster and exhibited only poor identity to the partial sequence obtained in this study. Even though the polypeptide showed the same 8-amino acid insertion between residues 343 and 344, the first large insertion between amino acid residues 152 and 153 was missing (data not shown). However, subsequent 16sRNA analysis approved the genotype as *T. phaeum* strain PB and confirmed the DNA purity used for PCR. The question therefore arose as to whether *T. phaeum* might harbor two *fhs* alleles, as found in *T. acetatoxydans* and *C. ultunense*, which might not be targeted by one and the same degenerated primer pair. Specific primers intended to target the different partial sequences of *T. phaeum* were designed, but only the partial *fhs* gene obtained by the 3-SAO*fhs* primer pair was recovered (data not shown).

### *Tepidanaerobacter acetatoxydans* FTHFS1 and FTHFS2 were functionally active, but only FTHFS1 was expressed

**Figure 5:**
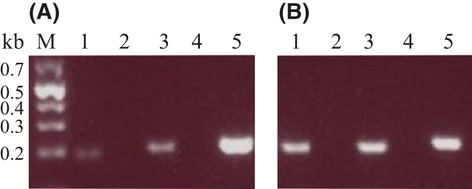
*Fhs* expression pattern in response to heterotrophic growth conditions producing acetate (A) and to syntrophic growth conditions consuming acetate (B). (1) *Tepidanaerobacter acetatoxydans* *fhs*1; (2) *T. acetatoxydans* *fhs*2; (3) *Clostridium ultunense* *fhs*1; (4) *Clostridium ultunense* *fhs*2; and (5) *Syntrophaceticus schinkii*.

The interesting finding of phylogenetically distinguished *fhs* genes harbored by one strain raised the question of which *fhs* gene is expressed in those strains harboring two alleles. To investigate this, mRNA samples from a coculture containing *C. ultunense*, *T. acetatoxydans*, and *S. schinkii* growing syntrophically with a hydrogenotrophic methanogen on acetate were compared against mRNA samples of the respective pure culture growing heterotrophically on lactate, glucose, and betaine, respectively. No differences in mRNA expression pattern were observed (Fig. 5). The *fhs*2 gene of both *T. acetatoxydans* and *C. ultunense* was found not to be expressed in either type of culture. However, the respective *fhs*1 gene was clearly expressed. The *fhs* gene of *S. schinkii* was expressed in both coculture and pure culture.

**Figure 6:**
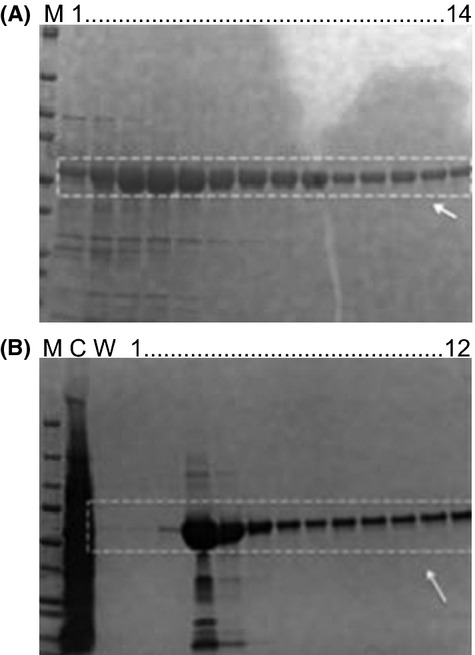
SDS-PAGE of His-tagged FTHFS1 (A) and FTHFS2 (B) of *Tepidanaerobacter acetatoxydans*. Purification was carried out by TALON metal-affinity chromatography as described under Experimental Procedures. M, molecular weight markers; C, cell extract; W, washing step; lane 1–14 (A) and lane 1–12 (B) = elution steps. Respective His-tagged FTHFS is framed and marked by an arrow.

Moreover, both FTHFS of *T. acetatoxydans* were purified to homogeneity and shown to be active as His-tag fusion proteins. The SDS-PAGE migration behavior was according to the deduced molecular weight of 60 kDa for a monomer (Fig. 6A and B). In addition, protein identity was confirmed by mass spectrometric peptide mapping. The specific activity of FTHFS1 was with 23 U/mg protein, approximately three times lower than the specific activity of FTHFS2, with 60 U/mg protein.

### The *fhs*1 gene of *T. acetatoxydans* is surrounded by genes belonging to both the methyl and carbonyl branch of the W–L pathway forming a putative operon

**Figure 7:**
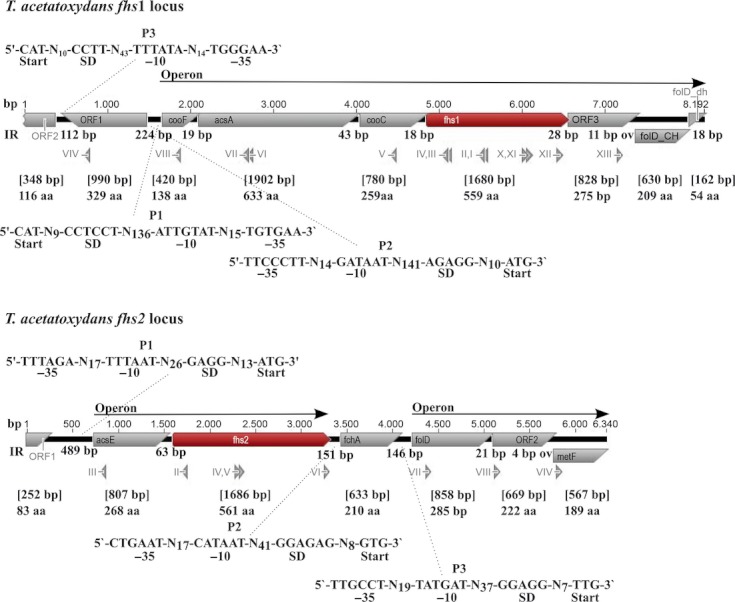
Gene organization of the *fhs* loci of *Tepidanaerobacter acetatoxydans* identified by genome walking. Genome walk primers as listed in Table 2 are depicted in Latin numerals; direction of arrows reflects upstream or downstream walking. Gene sizes and deduced polypeptide lengths are given in bp and aa, respectively. P1/P2/P3, putative promoter sites; IR, intergenic regions given in bp; SD, Shine–Dalgarno sequence; aa, amino acid residues; bp, base pairs. Enzyme restriction sites are not shown for the sake of clarity. ORF, open reading frame; *cooC*, Ni-insertion protein required for CODH maturation; *metF*, methylenetetrahydrofolate reductase; *acsE*, methyltransferase; *cooS*, CODH subunit; *fchA*, methenyltetrahydrofolate cyclohydrolase; *folD*, methylenetetrahydrofolate dehydrogenase; *fhs*, formyltetrahydrofolate synthetase; and *cooF*, Fe–S protein.

To obtain more information about the gene structure surrounding the respective *fhs* gene, we continued genome walking upstream and downstream. In the case of *T. acetatoxydans fhs*1, the flanking genome segments were analyzed up to 8.2 kb, encompassing eight further ORFs, while in the case of *T. acetatoxydans fhs*2, the flanking genome segments were analyzed up to 6.3 kb, encoding six more ORFs. The location, size, and deduced polypeptide length of these are summarized in Figure 7. Amplification of large fragments from each *T. acetatoxydans fhs* locus confirmed the accurate assembly and the existence of two clearly distinguished loci (see Experimental Procedures). Within the first *fhs* locus, an ORF designated *acsA* was identified upstream to *fhs*1 encoding a putative carbon monoxide dehydrogenase (CODH), the key enzyme of the carbonyl branch of the W–L pathway (Fig. 1). Adjacent downstream to *acsA*, a gene for a putative cobyrinic acid a,c-diamide synthase was found. The function of this enzyme has been described as essential for maturation of the Ni center of CODH (Kerby et al. 1997), and was named *cooC*. The deduced polypeptide of the third ORF (*cooF*) located upstream of *acsA* showed high similarity to iron–sulfur cluster-binding proteins. An ORF (ORF1) encoding a putative oxidoreductase was found to be divergently orientated to the *fhs*1 cluster. Analysis of the deduced amino acid sequence of the three ORFs adjacent downstream to *fhs*1 identified genes for two more putative enzymes of the W–L pathway: a methenyltetrahydrofolate cyclohydrolase designated *fchA* and a partial methylenetetrahydrofolate dehydrogenase designated *folD* (Fig. 1). The third ORF (ORF3), when blasted against the nonredundant protein database, showed low similarities to proteins with unknown function (as did ORF2) and therefore could not be allocated functionally. Two putative promoter sequences P1 and P2 (Fig. 7) were found to be arranged as face-to-face promoters (Beck and Warren 1988) within the intergenic region flanked by *cooF* and the divergently transcribed ORF1. As the intergenic regions located downstream of *cooF* are rather short and exhibited no further predictable promoter elements, the *fhs*1 locus was considered a putative operon. It was possible to predict a putative third promoter sequence (P3) at a distance of 57 bp from the start codon of ORF2.

### The *fhs*2 gene of *T. acetatoxydans* is embedded in genes belonging to the methyl branch of the W–L pathway

The five ORFs clustered with the second *fhs* were found to be organized into two putative operons (Fig. 7), separated by a single gene adjacent to its own putative promoter (P2). Interestingly, the *fhs*2 locus also possesses genes encoding a putative methenyltetrahydrofolate cyclohydrolase (*fchA*) and a methylenetetrahydrofolate dehydrogenase (*folD*), but those were not organized within one operon as seen for the *fhs*1 locus. Moreover, it proved possible to allocate two more ORFs to the W–L pathway: a putative methylenetetrahydrofolate reductase (*metF*) and a methyltetrahydrofolate methyltransferase (*acsE*) (Fig. 1). *FolD* and *metF* were found to be organized in one operon adjacent to a putative promoter sequence (P3) separated by an ORF (ORF2) that could not be allocated functionally. The second operon consisting of the putative promoter sequence P1, the methyltransferase gene *acsE*, and the second *fhs* gene was flanked upstream by a large AT-rich intergenic region of 489 bp, followed by a partial ORF (ORF1) that could not be related to any protein function.

All ORFs within the *fhs*1 locus were found to have an AUG start codon, whereas *fchA*, *folD* and ORF2 belonging to the *fhs*2 locus exhibited the alternative start codons GUG, UUG, and UUG, respectively. The corresponding Shine–Dalgarno sequences were identified within a distance of 7 bp to 11 bp from the respective initiation codons. No significant variation in GC content or average codon usage was observed within or between the different *fhs* clusters. However, all deduced proteins from the first *fhs* locus belonging to the W–L pathway were most similar to proteins from *T. oceani*, ranging from 60% to 85% identity, whereas the proteins from the second *fhs* locus were rather identical to proteins from *C. carboxidivorans* or *C. ljungdahlii* except for methylenetetrahydrofolate reductase and methyltransferase. The identities of the deduced polypeptides of *fhs*2, *fchA*, ORF2, and *folD* ranged from 63% to 79% compared with *C. carboxidivorans* and 62% to 79% compared with *C. ljungdahlii*. The methylenetetrahydrofolate reductase exhibited 74% identity to the protein of *Clostridium ragsdalii* or *C. difficile*, but still 69% identity to the protein of *C. carboxidivorans*. On the contrary, the methyltransferase was most similar (81% identity) to a protein of *Thermincola potens*.

Comparing the two FTHFS of *T. acetatoxydans*, 74% of the amino acid residues were identical and 86% were similar, whereas only 54% sequence identity and 75% similarity, respectively, were observed between the methenyltetrahydrofolate cyclohydrolases of these two loci. The putative oxidoreductase clustering together with the *fhs*1 operon showed no similarities to any of the oxidoreductases of the bacteria mentioned above. Instead, it exhibited 60% identity to a protein of the *Geobacillus* species WCH70.

### The *fhs* genes identified in *S. schinkii* and *C. ultunense* were located separately from other genes of the W–L pathway

**Figure 8:**
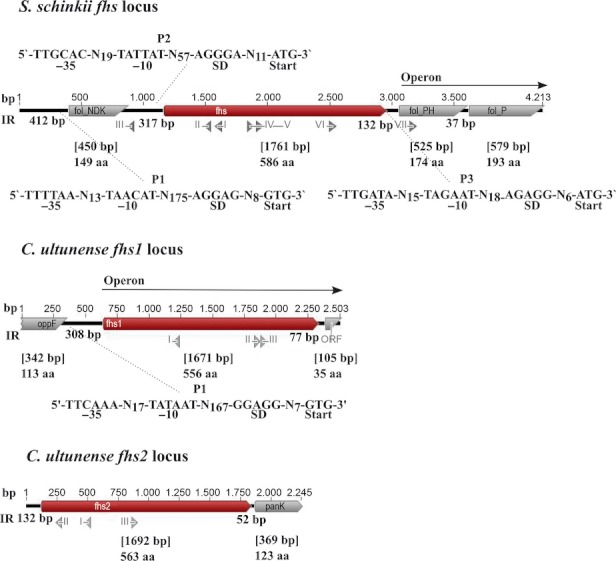
Gene organization of the *fhs* loci of *Syntrophaceticus schinkii* and *Clostridium ultunense* identified by genome walking. Genome walk primers as listed in Table 2 are depicted in Latin numerals; direction of arrows reflects upstream or downstream walking. Gene sizes and deduced polypeptide lengths are given in bp and aa, respectively. P1/P2/P3, putative promoter sites; IR, intergenic regions given in bp; SD, Shine–Dalgarno sequence; aa, amino acid residues; bp, base pairs. Enzyme restriction sites are not shown for the sake of clarity. ORF, open reading frame; *fol_PH*, phosphohydrolase; *fol_P*, dihydropteroate synthase; *fol_NDK*, nucleoside diphosphate kinase; *oppF*, oligopeptide/dipeptide ABC transport system; *fhs*, formyltetrahydrofolate synthetase; and *panK*, pantothenate kinase TypIII.

No further genes belonging to the W–L pathway could be identified within the adjacencies to the *fhs* genes of *S. schinkii* and *C. ultunense* (Fig. 8). A putative metal-dependent phosphohydrolase and a partial dihydropteroate synthase encoded by genes designated as *fol_PH* and *fol_P*, respectively, were identified downstream of the *S. schinkii fhs* gene. The short 37-bp intergenic region and a putative promoter sequence (P3) identified upstream of *fol_PH* suggested an operon consisting of at least *fol_PH* and *fol_P*. A putative nucleoside diphosphate kinase gene designated *fol_NDK* was found located upstream of *fhs* and separated by a large intergenic region of 317 bp, including a putative promoter sequence (P2) for *fhs*. The dihydropteroate synthetase catalyzes the condensation of *p*-aminobenzoic acid and 6-hydroxymethyl-7,8-dihydropterin pyrophosphate in the de novo biosynthesis of folate (de Crécy-Lagard et al. 2007). As this pathway requires both phosphoryl group transfer reactions and phosphoryl group hydrolysis, the putative *fol_PH* and *fol_NDK* genes might also belong to the folate biosynthesis pathway. *Fol_NDK* and *fol_P* were found to have GTG instead of ATG as their start codon. Putative Shine–Dalgarno sequences were found within a distance of 6–10 bp. No differences in codon usage or GC content were observed. The deduced polypeptides of *fol_NDK*, *fol_PH*, and *folP* of *S. schinkii* were most identical to proteins of *Pelotomaculum thermopropionicum* (74%), *T. potens* (54%), and *Desulfotomaculum kuznetsovii* (60%), respectively. The respective similarities were 85%, 73%, and 75%.

The *fhs*1 gene of *C. ultunense* clustered together with a gene encoding the ATPase subunit of a putative oligopeptide/dipeptide ABC transport system (*oppF*) separated by a large intergenic region of 308 bp. Furthermore, a partial ORF was found located downstream of *fhs*1 encoding a partial polypeptide consisting of 35 amino acid residues. An operon structure seems to be likely, due to the fact that putative promoter elements were only found upstream of the *fhs* gene and the short intergenic region between *fhs* and *oppF*. The GC content and the codon usage pattern were equal within this cluster, but GTG and TTG were found as start codons for *fhs* and the adjacent ORF, respectively. The deduced polypeptide of *oppF* exhibited 89% similarity and 64% identity to an oligopeptide ABC transporter of *Alkaliphilus oremlandii*. The partial polypeptide deduced from the ORF showed features of the helix–turn–helix superfamily, but could not be further allocated. Analysis of the second *fhs* locus of *C. ultunense* encountered difficulties because no pure upstream fragment larger than 132 bp could be amplified. The fragment obtained seemed to represent an intergenic region without any regulatory or promoter sequences. The downstream-located ORF designated *panK* encodes a partial putative pantothenate kinase Type III, which catalyzes the phosphorylation of pantothenate as the first step of its conversion to coenzyme A. The short intergenic region between *fhs*2 and *panK* did not show any promoter elements, which might point to an operon structure. The blastp search algorithm identified *Bacillus coagulans* as the closest relative to *panK*, sharing 61% sequence identity and 76% similarity. Compared with the *fhs*1 locus, a remarkable discrepancy in GC content was observed. Within the *fhs*1 locus, the GC content ranged between 28.6% and 35.5%, whereas within the *fhs*2 locus, it varied from 49.1% to 51.2%. Moreover, distinct changes in codon usage were detected on comparing the two *fhs* loci of *C. ultunense*.

## Discussion

**Figure 9:**
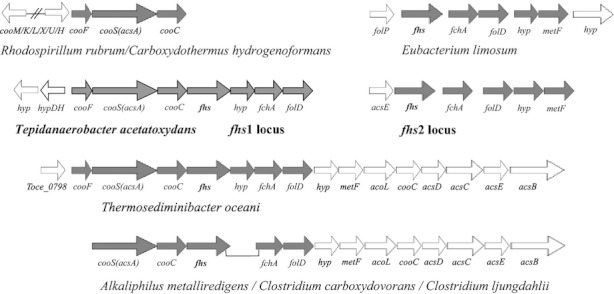
Comparison of the *Tepidanaerobacter acetatoxydans* *fhs* clusters to the *fhs* gene clusters of *Alkaliphilus metalliredigens*, *Clostridium carboxidivorans*, *C. ljungdahlii*, *Thermosediminibacter oceani*, and *Eubacterium limosum* and to the *coo* cluster of *Carboxydothermus hydrogenoformans* and *Rhodospirillum rubrum* involved in CO oxidation. *acsC*/*acsD*, corrinoid iron–sulfur protein; *cooC*, Ni-insertion protein required for CODH maturation; *metF*, methylenetetrahydrofolate reductase; *acsE*, methyltransferase; *acsB*, acetyl-CoA synthase subunit; *cooS/acsA*, CODH subunit; *hyp*, hypothetical protein; *fchA*, methenyltetrahydrofolate cyclohydrolase; *folD*, methylenetetrahydrofolate dehydrogenase; *folP*, dihydropteroate synthase; *fhs*, formyltetrahydrofolate synthetase; *acoL*, dihydrolipoamide dehydrogenase; *cooF*, Fe–S protein; *cooM/K/L/X/U/H*, six-subunit [NiFe] hydrogenase; *Toce_0798*, amidohydrolase; and *hypDH*, putative dehydrogenase.

To our knowledge, only five complete genomes of acetogenic bacteria, covering *T. oceani* (Pitluck et al. 2010), *C. ljungdahlii* (Köpke et al. 2010), *M. thermoacetica* (Pierce et al. 2008), *Eubacterium limosum* (Roh et al. 2011), and *A. woodii* (Poehlein et al. 2012) have been sequenced and all genes encoding the enzymes of the W–L pathway have been annotated (The last one was published quite recently and therefore not analyzed comparatively.). In addition, the genome of the strictly CO-utilizing bacterium *C. hydrogenoformans* (Wu et al. 2005), the metal-reducing bacterium *A. metalliredigens* (Ye et al. 2004), and the W–L pathway gene cluster of the CO-utilizing *C. carboxidivorans* (Bruant et al. 2010) have been reported. The putative operon identified here within the *T. acetatoxydans fhs*1 locus was found to be identical in gene organization to the W–L pathway gene cluster of *T. oceani* (Fig. 9) and most identical concerning gene identities. As the 16sRNA genes showed 89% sequence identity, a common ancestor is rather more likely than horizontal gene transfer. Moreover, the locus structure seems to be highly conserved when compared with the *fhs* gene clusters of *C. ljungdahlii*, *A. metalliredigens*, and *C. carboxidivorans* (Fig. 9), leading us to speculate that the *T. acetatoxydans fhs*1 locus might continue in the same way encoding all proteins of the W–L pathway. Interestingly, two additional genes designated *cooF* and *hyp* were found to be part of the operon in *T. acetatoxydans fhs*1 and *T. oceani*, but not in the other three bacteria species mentioned. *CooF* homologs, analyzed by the blastp search algorithm, were mainly found in sulfate reducers and CO oxidizers, but seemed not to be common in acetogens. Moreover, the organization of *cooF* showed similarities to the *Coo* regulon of *Rhodospirillum rubrum* (Singer et al. 2006) and to *C. hydrogenoformans* (Wu et al. 2005), where *cooF*, *cooS,* and *cooC* are arranged in the same way as in *T. acetatoxydans* and *T. oceani* (Fig. 9). These bacteria can grow using carbon monoxide as the sole carbon and energy source, producing H_2_ and CO_2_ by using homolog enzymes of the carbonyl branch of the W–L pathway. Here, CooF is proposed to be the electron transfer mediator between CODH and the respective CO-induced hydrogenases, coupling the oxidation of CO to CO_2_ with H_2_ evolution. In *R. rubrum*, a gene cluster 460 bp upstream of the *Coo* regulon encodes a six-subunit [NiFe] hydrogenase that catalyzes H_2_ evolution, likewise in *C. hydrogenoformans*. In *T. acetatoxydans*, the divergently orientated putative oxidoreductase located upstream of the *fhs*1 cluster might have functional similarities to the [NiFe] hydrogenase complex mentioned above. This clearly distinguished *T. acetatoxydans* from *T. oceani*, where the upstream structure adjacent to *cooF* does not show a divergently orientated ORF. In consideration of the finding that no homolog proteins could be identified in *T. oceani* or in any other acetogenic bacteria, the upstream region might have been modified by horizontal gene transfer. This assumption is supported by the finding of a DNA fragment identified farther upstream of the *T. acetatoxydans fhs*1 locus encoding a partial sequence of a putative transposase belonging to the IS4 family (data not shown). Interestingly, the hypothetical protein (*hyp* or ORF3) unique within the *fhs* cluster of *T. oceani* and *T. acetatoxydans* shared, with few exceptions only, identities with hypothetical proteins of archaea including members of the families *Methanosaetaceae* and *Methanosarcinaceae*, some of which are known to be partly aceticlastic methanogens. The fact that no function was allocatable might point to an unknown regulatory protein. The abundance of repetitive and palindromic sequences (data not shown) identified within the intergenic region of the *fhs* operon and the divergently orientated putative oxidoreductase indicates a rather strongly regulated operon. The putative face-to-face promoters P1 and P2 encompassing several of those putative regulatory sites suggest simultaneous up- and downregulation of the *fhs* operon and the putative oxidoreductase, indicating a function of the latter connected to acetogenesis and/or acetate oxidation. However, the possibility cannot be excluded that other sequences may function as weak promoter sites resulting in back-to-back promoters, which might allow alternative regulatory mechanisms. In conclusion, an operon encoding enzymes of the entire W–L pathway might be one of the essential adaptations needed for the energy-limited syntrophic lifestyle, as presumed for the methylmalonyl-CoA pathway operon of the syntrophic propionate oxidizer *P. thermopropionicum* (Kato and Watanabe 2010), due to increasing the transcriptional efficiency.

The second *fhs* locus seems to be unique to *T. acetatoxydans*: None of the bacteria mentioned above (or any other acetogen characterized) have been found to harbor a second *fhs* gene. The cluster organization mainly shows similarities to the cluster found in *E. limosum* regarding gene composition and operon structure (Fig. 9). No further genes belonging to the W–L pathway were found adjacent to the methylenetetrahydrofolate reductase gene (*metF*) in *E. limosum* and the same situation may also occur in *T. acetatoxydans*. As none of the ORFs showed any similarities to genes of *T. oceani* or to any more closely related bacteria, the additional gene set might have been achieved once by horizontal gene transfer. Although both FTHFSs were shown to be active and the second locus showed a functional gene organization, the mRNA expression studies suggest that only the *T. acetatoxydans fhs*1 operon is functional under heterotrophic and syntrophic growing conditions. However, depending on the habitat, there may be other growing conditions where the *fhs*1 operon and subsequently the W–L pathway genes become repressed. As indicated by the findings reported above, the *T. acetatoxydans fhs*1 cluster seems to be regulated rather than expressed constitutively. It has been shown for *A. woodii*, when growing heterotrophically in coculture with a hydrogentrophic methanogen, that the electron flow is directed toward hydrogenases, evolving H_2_ directly consumed by the methanogens and bypassing the W–L pathway (Winter and Wolfe 1980). Likewise in *P. productus,* electrons are directed away from the W–L pathway when the CO_2_ partial pressure becomes low (Misoph and Drake 1996). Under these circumstances, the activities of FTHFS, methylenetetrahydrofolate dehydrogenase, methenyltetrahydrofolate cyclohydrolase, methyltransferase, and methylene reductase are still essential for the one-carbon metabolism. It is conceivable that the second *fhs* locus represents an alternative gene cluster supplying the cells with C1 units at different oxidation levels, helping out when the first locus is becoming repressed. This hypothesis is supported by the fact that only proteins belonging to the methyl branch were found to be encoded by the second *fhs* locus, as analyzed and compared with *E. limosum*. Moreover, the three putative promoter sequences suggest more flexible up- and downregulation compared with the first *fhs* locus, which might enable the cells to respond to the demand of C1 units at different oxidation levels in a more efficient way.

In contrast, the *fhs* genes identified in *S. schinkii* and *C. ultunense* were found to be located separately from other genes of the W–L pathway, as is the case in *M. thermoacetica*. Interestingly, the *fhs*1 gene identified in *C. ultunense* was found to be mostly similar to that of purine-fermenting *Clostridia*, which do not harbor the W–L pathway genes, but instead use the reverse reaction of FTHFS to generate ATP during purine fermentation as the only energy source. In other words, this group recruits the same enzyme function as needed by SAOB, when running the W–L pathway in an oxidative direction. The operon structure described here distinguishes *C. ultunense* from *M. thermoacetica* insofar as a putative regulatory protein might be encoded downstream of *fhs*1 allocated by a putative helix–turn–helix motif known from DNA-binding proteins. Accordingly, palindromic sequences were found upstream of the promoter, suggesting a putative transcription activator recognition site (data not shown). Unfortunately, to date, none of the genomes from purinolytic bacteria, which might have given us additional information, have been published. The second *fhs* gene identified in *C. ultunense* did not show any similarities to known acetogens. The difference in GC content and codon usage points clearly to a horizontal gene transfer event. Moreover, only *fhs*1 was found to be expressed under both acetate-producing and acetate-consuming conditions. A similar alternative regulation mechanism as suggested for the *T. acetatoxydans fhs* clusters might be possible, but on the other hand, the differences in DNA character and codon usage might reduce or even prevent efficient transcription and translation.

In both *S. schinkii* and *M. thermoacetica*, the respective *fhs* gene seems to be associated with an operon encoding enzymes necessary for the *de novo* synthesis of tetrahydrofolate, the coenzyme of FTHFS. The *S. schinkii fhs* gene was found to be grouped closely to sulfate-reducing bacteria. These bacteria use the W–L pathway in the oxidative way when fermenting organic components and direct the electrons through the W–L pathway to sulfate and regain energy (Fig. 1). The genome of the closest sulfate-reducing relative (*Desulfotomaculum orientis*) encodes two *fhs* genes lying apart from each other and from other W–L pathway genes. It is possible that a putative second *fhs* gene cannot be detected in *S. schinkii* using this primer pair. Otherwise, the mRNA expression study showed the identified *fhs* gene as clearly expressed in both types of growing conditions. Interestingly, two large insertions were found that distinguished the FTHFS of *S. schinkii* and *T. phaeum* clearly from other known FTHFS and connected them to the sulfate reducers. Based on the crystal structure of FTHFS from *M. thermoactica* (Radfar et al. 2000), these large insertions might dramatically alter the region, which is involved in tetramerization. All bacterial FTHFS purified to date have been reported to be functional as homotetramers (Scott and Rabinowitz 1967; Brewer et al. 1970; MacKenzie and Rabinowitz 1971; O'Brien et al. 1976; Marx et al. 2003). However, none of these has been isolated from a sulfate reducer. Thus, additional residues might change the quaternary structure toward a more stable tetramer or even a functional dimer affecting the overall enzymatic activity of the pathway Interestingly, another acetogen, *A. longum*, was found to be aligned within the sulfate reducers (Fig. 3), closely related to the SAOB *S. schinkii* and *T. phaeum*. This species was first isolated from the gut of wood-feeding termites (Kane and Breznak 1991), but to date, none of the gut commensals have been reported to be syntrophic acetate oxidizers.

Finally, it must be stated that the phylogenetic diversity observed here for FTHFS from the SAOB and the allelomorphism demand reevaluation of the degenerated primer pairs used in acetogenic community studies with respect to their specificity for SAOB and associated conclusions regarding species abundance.
